# Preparedness of the Ghana Health Service for field epidemiology and applied biostatistics: a systematic review of infectious disease surveillance, outbreak investigation methodologies, and statistical modeling capacities in resource-limited settings

**DOI:** 10.3389/fpubh.2026.1814212

**Published:** 2026-09-16

**Authors:** Victor Luckyboy Dzramado, Obed U. Lasim, William Wilberforce Amoah, Samuel Antwi, Joana Edem Koto, Doris Hagan

**Affiliations:** 1 Department of Biostatistics, Cape Coast Teaching Hospital, Cape Coast, Ghana; 2 Department of Health Information Management, School of Allied Health Sciences, University of Cape Coast, Cape Coast, Ghana; 3 Department of Emergency and Critical Care Nursing, School of Nursing and Midwifery, Kwame Nkrumah University of Science and Technology, Kumasi, Ghana; 4 School of Public Health, University of Ghana, Legon, Accra, Ghana; 5 Kintampo Health Research Centre, Kintampo, Bono East Region, Ghana; 6 Department of Public Health Nursing, School of Nursing and Midwifery, Kwame Nkrumah University of Science and Technology, Kumasi, Ghana

**Keywords:** capacity building, FELTP, field epidemiology, Ghana Health Service, IDSR, outbreak investigation, preparedness and response, surveillance systems

## Abstract

**Introduction:**

Infectious disease outbreaks continue to threaten global health security, with resource-limited settings facing disproportionate challenges in surveillance, outbreak investigation, and applied biostatistical capacity. The WHO International Health Regulations 2005 framework defines preparedness through measurable core capacities, and seminal multi-country analyses of IHR State Party Self-Assessment Annual Reporting data from 182 and 186 countries consistently show that sub-Saharan African countries report the largest preparedness gaps globally. Ghana, despite hosting the first Field Epidemiology and Laboratory Training Programme in West Africa, has not previously been the subject of a comprehensive synthesis of its preparedness across all relevant IHR domains. This review therefore addresses a single integrated question: to what extent is the Ghana Health Service prepared for field epidemiology and applied biostatistics, as assessed through surveillance system performance, outbreak investigation capacity, workforce development, laboratory infrastructure, and statistical modeling capacities aligned with WHO IHR core capacity benchmarks?

**Methods:**

This systematic review followed PRISMA 2020 guidelines and was prospectively registered on PROSPERO (CRD420261299788). Searches were conducted in PubMed, African Index Medicus, AJOL, and Google Scholar, supplemented by grey literature, covering January 2000 to February 2026. Two reviewers independently screened 328 unique records, assessed 75 full-text articles, and included 38 studies with Ghana-specific disaggregated data. Quality was assessed using design-specific tools with transparent reconciliation into low, moderate, and high risk-of-bias categories. Narrative synthesis was the principal analytic approach.

**Results:**

Surveillance completeness ranged from 71% to 94% (median 82%); timeliness ranged from 48% to 91% (median 76%), with regional performance substantially exceeding district performance. The GFELTP produced 420 graduates from 2007 to 2017, representing 45% of WHO-benchmarked workforce requirements. Outbreak response times improved from 14 to 3 days for comparable outbreaks. Laboratory capacity remained concentrated in 2 to 6 sentinel sites. No included study examined biostatistical modeling capacity. The 2017 WHO Joint External Evaluation rated overall IHR capacity at 67%.

**Discussion:**

Ghana demonstrates advancing but incomplete preparedness. Priority interventions should address workforce expansion, peripheral surveillance strengthening, laboratory decentralization, and indigenous biostatistical capacity development.

**Systematic review registration:**

https://www.crd.york.ac.uk/PROSPERO/view/CRD420261299788, identifier: CRD420261299788.

## 1 Introduction

### 1.1 Global context and seminal work on IHR core capacities

Infectious disease outbreaks produce devastating health, social, and economic consequences that disproportionately affect resource-limited settings (1, 2). The 2014 to 2016 West African Ebola epidemic, recurrent cholera outbreaks across sub-Saharan Africa, cerebrospinal meningitis epidemics in the meningitis belt, the COVID-19 pandemic, and the ongoing Ebola outbreak in the Democratic Republic of Congo collectively expose fundamental preparedness and response capacity gaps, particularly in low- and middle-income countries (3, 4). The Ebola crisis alone cost affected West African economies over USD 2 billion within two years, underscoring the imperative for robust, sustained preparedness infrastructure (5).

The World Health Organization International Health Regulations 2005 (IHR 2005) provide the global legal and operational framework for infectious disease surveillance and response, defining preparedness through 19 core capacity domains spanning prevention, detection, and response (6). A growing body of seminal multi-country research has used IHR State Party Self-Assessment Annual Reporting (SPAR) and Joint External Evaluation (JEE) data to characterize national preparedness comparatively. Kandel and colleagues, in a foundational analysis of IHR annual report data from 182 countries published in the context of the early COVID-19 outbreak, demonstrated that only approximately half of countries had operational readiness capacities to manage emerging high-impact respiratory pathogens, with significant gaps concentrated in sub-Saharan Africa (7). Eze and colleagues, in a more recent assessment of epidemic preparedness and response capacity across 186 countries using SPAR submissions from 2018 to 2022, found that although capacities improved marginally over five years, approximately one-third of countries still had inadequate functional capacity at national and sub-national levels, with sub-Saharan African countries reporting the most pronounced gaps across all five capacity indexes (8). Gostin and colleagues, in their critical examination of the 2024 amendments to the International Health Regulations, argue that even with the recent regulatory revisions adopted by the 77th World Health Assembly, the underlying structural inequities in preparedness financing and capacity persist across low- and middle-income settings, leaving regions such as West Africa particularly vulnerable to recurrent and emerging threats (9).

This consistent finding across the seminal IHR-based literature, that sub-Saharan African countries face the largest preparedness gaps, provides the framing within which Ghana-specific evidence must be interpreted. Ghana operates at the intersection of substantial endemic infectious disease burden and persistent structural resource constraints, and serves as a regional hub for Anglophone West African field epidemiology training. A focused synthesis of Ghana's evidence base therefore informs both national policy and broader sub-regional preparedness investment decisions.

Field epidemiology, defined as the application of epidemiological methods to investigate and control health problems in defined populations, forms the operational core of outbreak detection and response (10). Countries with established Field Epidemiology Training Programmes demonstrated measurably superior outbreak control during the Ebola epidemic compared with countries lacking such capacity (11). Applied biostatistics and statistical modeling capabilities prove similarly critical for analyzing surveillance data, predicting epidemic trajectories, informing resource allocation, and evaluating programme effectiveness (12). The COVID-19 pandemic confirmed the necessity of indigenous epidemic modeling capacity for time-sensitive policy decision-making, yet such capabilities remain largely absent across resource-limited settings (13).

### 1.2 The Ghana context

Ghana, a lower-middle-income West African nation with approximately 33 million inhabitants across 16 regions, operates a decentralized health system under the Ministry of Health, with the Ghana Health Service serving as the principal implementer of public health services (14). The country faces substantial infectious disease burden, including endemic malaria accounting for 38% of outpatient visits, recurrent cholera in coastal and urban areas, cerebrospinal meningitis in northern regions, yellow fever, vaccine-preventable diseases, and emerging infections including viral hemorrhagic fevers and COVID-19 (15, 16).

Ghana has implemented the WHO-supported Integrated Disease Surveillance and Response (IDSR) strategy since 2003, featuring weekly disease reporting through the District Health Information Management System 2 (DHIMS2) electronic platform (17, 18). The Ghana FELTP, established in 2007 at the University of Ghana School of Public Health with United States Centers for Disease Control and Prevention support, was the first FELTP established in West Africa and serves as a sub-regional training hub for Anglophone countries (19, 20).

Despite these substantial investments, comprehensive systematic synthesis of Ghana's preparedness across surveillance systems, outbreak investigation capacity, workforce adequacy, laboratory infrastructure, and biostatistical capabilities has not previously been undertaken. Previous evaluations have focused on individual components in isolation without aggregating the broader evidence base (21, 22). This review addresses that gap, providing the first comprehensive Ghana-specific synthesis of field epidemiology preparedness across all WHO IHR core capacity domains relevant to field epidemiology and applied biostatistics.

### 1.3 Choice of study design (systematic review)

We considered whether the appropriate study design for this body of evidence was a systematic review or a scoping review, given the breadth of preparedness domains under consideration. A scoping review is most appropriate when the objective is to map the extent, range, and nature of evidence on a broad topic without explicit quality appraisal and without quantitative summary of effects (23). A systematic review is most appropriate when the objective is to answer a focused question, apply transparent inclusion criteria, conduct formal critical appraisal of included studies, and synthesize findings against pre-specified outcome domains (24). This review was designed and conducted as a systematic review for three explicit reasons. First, the research question was tightly focused on a single national health system (the Ghana Health Service) and a defined set of preparedness outcome domains aligned with WHO IHR core capacities, rather than broadly mapping the field. Second, formal risk-of-bias assessment was applied to all 38 included studies using design-specific tools, with explicit reconciliation rules into low, moderate, or high categories. Third, all reporting in this review conforms to PRISMA 2020 guidelines for systematic reviews. While certain broad descriptive elements of the synthesis share features with scoping reviews, the focused question, formal appraisal, and PRISMA conformance situate this work appropriately as a systematic review. We acknowledge the legitimate alternative framing raised in peer review and have made the rationale for the chosen design explicit here.

### 1.4 Primary research question and objectives

#### 1.4.1 Primary research question

To what extent is the Ghana Health Service prepared for field epidemiology and applied biostatistics, as assessed through performance metrics aligned with WHO IHR core capacities in surveillance systems, outbreak investigation, workforce development, laboratory infrastructure, and statistical modeling capacity?

#### 1.4.2 Secondary objectives

1. Assess the performance of Ghana's infectious disease surveillance systems, including the IDSR platform and disease-specific surveillance programmes.
2. Evaluate outbreak investigation methodologies, response capacity, and temporal trends in outbreak response performance.
3. Examine GFELTP outcomes including graduate numbers, deployment patterns, and contribution to outbreak response.
4. Assess laboratory capacity for infectious disease diagnostics and surveillance support across pathogen types.
5. Evaluate health system preparedness against WHO IHR core capacity benchmarks.
6. Identify evidence regarding biostatistical and epidemic modeling capabilities and characterize any evidence gaps.

## 2 Methods

### 2.1 Protocol and registration

This systematic review was conducted following the PRISMA 2020 guidelines (24). The completed PRISMA 2020 checklist is provided as Supplementary File 1. The protocol was prospectively registered with the International Prospective Register of Systematic Reviews (PROSPERO) on February 3, 2026 (Registration Number: CRD420261299788), accessible at https://www.crd.york.ac.uk/PROSPERO/view/CRD420261299788. No substantial protocol deviations occurred during the conduct of the review.

### 2.2 Eligibility criteria (PICOS framework with Ghana-specific data requirement)

This review is specifically focused on the Ghana Health Service and Ghana's national public health system. Inclusion required that studies provide Ghana-specific data that could be disaggregated for analysis; multi-country studies were included only when Ghana-specific data were separately reported or extractable. Table 1 presents the complete PICOS eligibility framework applied throughout screening and data extraction, with the Ghana-specific disaggregation requirement explicitly stated.

**Table 1:** PICOS framework for study eligibility with Ghana-specific disaggregation requirement.

Component	Inclusion criteria	Exclusion criteria
Population	Ghana Health Service, Ghana's public health system, disease surveillance infrastructure in Ghana, GFELTP workforce, or population-level disease surveillance in Ghana. Multi-country studies were eligible only if Ghana-specific disaggregated data were reported or extractable.	Studies exclusively examining clinical management without surveillance or epidemiology components. Multi-country studies without Ghana-specific disaggregated data.
Interventions/exposures	Surveillance systems and IDSR implementation; outbreak investigation and response; GFELTP programme implementation; laboratory capacity for infectious disease; biostatistical capacity and epidemic modeling; IHR core capacity development.	Not applicable.
Comparators	Comparisons with WHO benchmarks, regional averages, or prior time periods were accepted but not mandatory.	Not applicable.
Outcomes (Primary)	(1) Surveillance system performance indicators (completeness, timeliness, sensitivity, positive predictive value, data quality); (2) Outbreak response metrics (detection time, response time, case fatality rates, control measure implementation).	Not applicable.
Outcomes (secondary)	(1) Workforce capacity indicators; (2) Laboratory capacity indicators; (3) Preparedness assessment scores (IHR core capacity and Joint External Evaluation findings).	Not applicable.
Study designs	Cross-sectional studies; cohort studies; case-control studies; mixed-methods studies; programme evaluations; surveillance system evaluations; outbreak investigation reports; technical reports and grey literature from WHO, GHS, WAHO, and AFENET.	Systematic reviews and meta-analyses (cited where relevant but not included as primary evidence); editorials and opinion pieces without primary data; conference abstracts without full text; duplicate publications.
Setting	Ghana or Ghana Health Service operations, with Ghana-specific data extractable from multi-country studies where applicable.	Studies not providing Ghana-specific data, or where Ghana-specific data could not be disaggregated.
Time period	January 2000 to February 2026.	Studies whose data collection fell outside the review's timeframe.
Language	English language publications.	Non-English publications.

### 2.3 Information sources and search strategy

Comprehensive searches were conducted across four electronic databases selected for coverage of African health research: PubMed/MEDLINE, African Index Medicus, African Journals Online (AJOL), and Google Scholar. Search terms were organized around three concept groups, namely geographic terms (Ghana, West Africa, Ghanaian), health system terms (Ghana Health Service, public health, health system), and field epidemiology terms (preparedness, surveillance, IDSR, field epidemiology, FELTP, GFELTP, outbreak, epidemic, pandemic, biostatistics, statistical modeling, laboratory capacity, IHR). Concepts were combined using Boolean AND operators; synonyms within concepts were combined with OR. Medical Subject Headings (MeSH) terms were used in PubMed where applicable. Table 2 presents the complete systematic summary of all search strategies including grey literature sources.

**Table 2:** Systematic summary of database search strategies and grey literature sources.

Source	Search date	Strategy summary	Records retrieved
PubMed/MEDLINE	15 Jan 2026	(Ghana[tiab] OR “West Africa”[tiab]) AND (“health service^*^”[tiab] OR “public health”[tiab] OR “Ghana Health Service”[tiab]) AND (preparedness[tiab] OR surveillance[tiab] OR “field epidemiology”[tiab] OR FELTP[tiab] OR GFELTP[tiab] OR IDSR[tiab] OR “outbreak investigation”[tiab] OR biostatistics[tiab] OR epidemiologist^*^[tiab] OR “laboratory capacity”[tiab]). Filters: 2000–2026, English.	186
African index medicus	16 Jan 2026	Search 1: Ghana AND (“field epidemiology” OR FELTP OR GFELTP OR “epidemiology training”). Search 2: “West Africa” AND (“field epidemiology” OR FELTP OR surveillance OR “outbreak response” OR preparedness) AND (Ghana OR ECOWAS OR WAHO).	18
African journals online (AJOL)	17 Jan 2026	Main query: Ghana AND (surveillance OR epidemiology OR outbreak OR preparedness OR FELTP OR IDSR OR biostatistics). Supplementary: Ghana AND biostatistics; Ghana AND “laboratory capacity”; Ghana AND epidemiologist; “Ghana Health Service” AND surveillance; Ghana AND cholera AND surveillance.	52
Google scholar	18–20 Jan 2026	Main query: Ghana (“field epidemiology” OR surveillance OR preparedness OR FELTP OR IDSR OR outbreak). First 200 results screened by title and abstract relevance.	200
Grey literature sources	Jan 2026	WHO Joint External Evaluation reports for Ghana; Ghana Health Service annual reports; GFELTP programme evaluations; IDSR assessment reports; outbreak investigation reports (cholera, CSM, COVID-19, measles, EVD); WAHO regional assessments; AFENET publications; institutional websites of GHS, University of Ghana School of Public Health, NMIMR, and CDC Africa.	15
Total (before deduplication)			**471**
Duplicates removed		Automated deduplication via Zotero (DOI, title, author matching) with manual verification	143
Total (unique records for screening)			**328**

Reference list hand-searching, forward citation searching, and direct author contact supplemented electronic database searches. All searches were completed by January 30, 2026.

### 2.4 Study selection process

Two reviewers (VD and OL) independently conducted title and abstract screening of all 328 unique records using Covidence systematic review software. A calibration exercise was conducted on 20 randomly selected records prior to formal screening, establishing inter-rater agreement of kappa = 0.82. Initial screening identified 250 records for exclusion. The primary reasons for exclusion at this stage were as follows. Geographic mismatch (study did not address Ghana or Ghana-specific data could not be disaggregated) accounted for 87 records. Wrong outcomes (study did not assess any of the predefined primary or secondary outcomes) accounted for 62 records. Wrong study type (study type fell outside the eligible designs listed in the PICOS framework) accounted for 41 records. Publication before January 2000, falling outside the review's timeframe, accounted for 23 records. Records identifying as systematic reviews accounted for 17 records, since systematic reviews were cited where relevant but not included as primary evidence. Non-English publications accounted for eight records. Late-identified duplicates accounted for 12 records. Seventy-eight records proceeded to full-text assessment.

Full-text retrieval was attempted for all 78 articles, and 75 were successfully retrieved (96.2%). Both reviewers independently assessed each full text, achieving 89.3% agreement (kappa = 0.76 at full-text stage). Eight discrepancies arose during full-text assessment, and these were resolved through consensus discussion with the third co-author serving as adjudicator where required. Final inclusion comprised 38 studies. Reasons for exclusion at the full-text stage were as follows. Fourteen articles were excluded for insufficient outcome data. Eight articles were excluded for wrong study design. Six articles were excluded because Ghana-specific data were not disaggregable from multi-country reporting. Four articles were excluded as duplicate data already reported in another included publication. Three articles were excluded as editorial pieces without primary data. Two articles were excluded because their data collection period fell outside the review's timeframe.

### 2.5 Data collection and extraction

A comprehensive data extraction form was developed covering study characteristics, population characteristics, methodological details, surveillance system performance metrics, outbreak investigation details, FELTP and workforce data, laboratory capacity indicators, biostatistical methods, and preparedness assessment scores. The form was pilot tested on five randomly selected included studies and refined accordingly. Both reviewers independently extracted data from all 38 included studies. Twelve data extraction discrepancies were identified and resolved by re-examination of source articles and consensus discussion.

### 2.6 Quality assessment

Quality assessment employed design-specific tools recognized in the relevant methodological literature. Two reviewers independently assessed quality of all 38 included studies, achieving kappa = 0.76 inter-rater agreement. Six disagreements were resolved through consensus discussion. The tools applied, their citations, and the reconciliation rules used to map their differing scoring scales into a single low, moderate, or high risk-of-bias categorization are presented in Table 3.

**Table 3:** Quality assessment tools, source citations, and reconciliation rules by study design.

Study design (*n*)	Tool applied	Source citation	Native scoring scale	Reconciliation rule into low/moderate/ high risk of bias
Cross-sectional studies (*n* = 15)	Newcastle-Ottawa Scale adapted for cross-sectional studies	Wells et al. (63) Ottawa Hospital Research Institute	0 to 10 stars across selection, comparability, and outcome domains	8 to 10 stars = low risk; 5 to 7 stars = moderate risk; 0 to 4 stars = high risk
Laboratory-based surveillance studies (*n* = 4)	Newcastle-Ottawa Scale adapted for cross-sectional studies	Wells et al. (63) Ottawa Hospital Research Institute	0 to 10 stars across selection, comparability, and outcome domains	8 to 10 stars = low risk; 5 to 7 stars = moderate risk; 0 to 4 stars = high risk
Surveillance system evaluations (*n* = 9)	CDC Updated Guidelines for Evaluating Public Health Surveillance Systems	German et al. (64) MMWR Recommendations and Reports	Narrative ratings across nine attributes (simplicity, flexibility, data quality, acceptability, sensitivity, PPV, representativeness, timeliness, stability), each rated as adequate, partially adequate, or inadequate	All nine attributes rated adequate = low risk; seven or eight attributes rated adequate = moderate risk; six or fewer attributes rated adequate = high risk
Programme evaluations (*n* = 8)	RE-AIM framework	Glasgow et al. (65) American Journal of Public Health	Narrative assessment across five domains (Reach, Effectiveness, Adoption, Implementation, Maintenance), each rated as strong, moderate, or weak	All five domains rated strong or moderate = low risk; three or four domains rated strong or moderate = moderate risk; two or fewer domains rated strong or moderate = high risk
Outbreak investigation reports (*n* = 10)	Outbreak Investigation Methodology Checklist adapted from WHO/CDC standards	WHO Guidelines for Surveillance and Outbreak Investigation (66)	Eight-item checklist (investigation methodology, case definition clarity, data completeness, analytical approach, laboratory confirmation, control measure documentation, reporting quality, lessons learned), each scored as present or absent	Seven or eight items present = low risk; five or six items present = moderate risk; four or fewer items present = high risk
Grey literature (*n* = 2)	AACODS checklist	Tyndall, Flinders University (67)	Six criteria (Authority, Accuracy, Coverage, Objectivity, Date, Significance), each rated as adequate or inadequate	All six criteria rated adequate = low risk; four or five criteria rated adequate = moderate risk; three or fewer criteria rated adequate = high risk

This explicit reconciliation framework ensured that the underlying differences in scoring scales across the five tools were translated transparently into a single comparable categorization, allowing readers to interpret the overall quality distribution presented in Section 3.3.

### 2.7 Data synthesis

Narrative synthesis was the primary analytic approach, selected because substantial heterogeneity in study designs, populations, outcome definitions, and time periods precluded quantitative meta-analysis (24). Extracted data were organized across six pre-specified thematic areas: (1) surveillance system performance, (2) outbreak investigation capacity, (3) GFELTP and workforce development, (4) laboratory capacity, (5) biostatistical capacity and epidemic modeling, and (6) health system preparedness and IHR core capacities. Quantitative data were tabulated across studies, and medians and ranges were calculated where comparable metrics were available. Subgroup analyses examined variation by health system level (national, regional, district, facility), geographic region, time period, and disease type. Meta-analysis would have been considered only if three or more studies had reported comparable outcomes using compatible methods and populations; this criterion was not met for any outcome domain.

## 3 Results

### 3.1 Study selection

The PRISMA 2020 flow diagram summarizes the search and selection process (Figure 1). Database and grey literature searches identified 471 records: PubMed (*n* = 186), African Index Medicus (*n* = 18), AJOL (*n* = 52), Google Scholar (*n* = 200), and grey literature (*n* = 15). After removing 143 duplicates, 328 unique records underwent title and abstract screening. Of these, 250 were excluded, leaving 78 records for full-text assessment, of which 75 were successfully retrieved. After full-text review, 37 articles were excluded, yielding 38 studies for inclusion in the synthesis.

**Figure 1:**
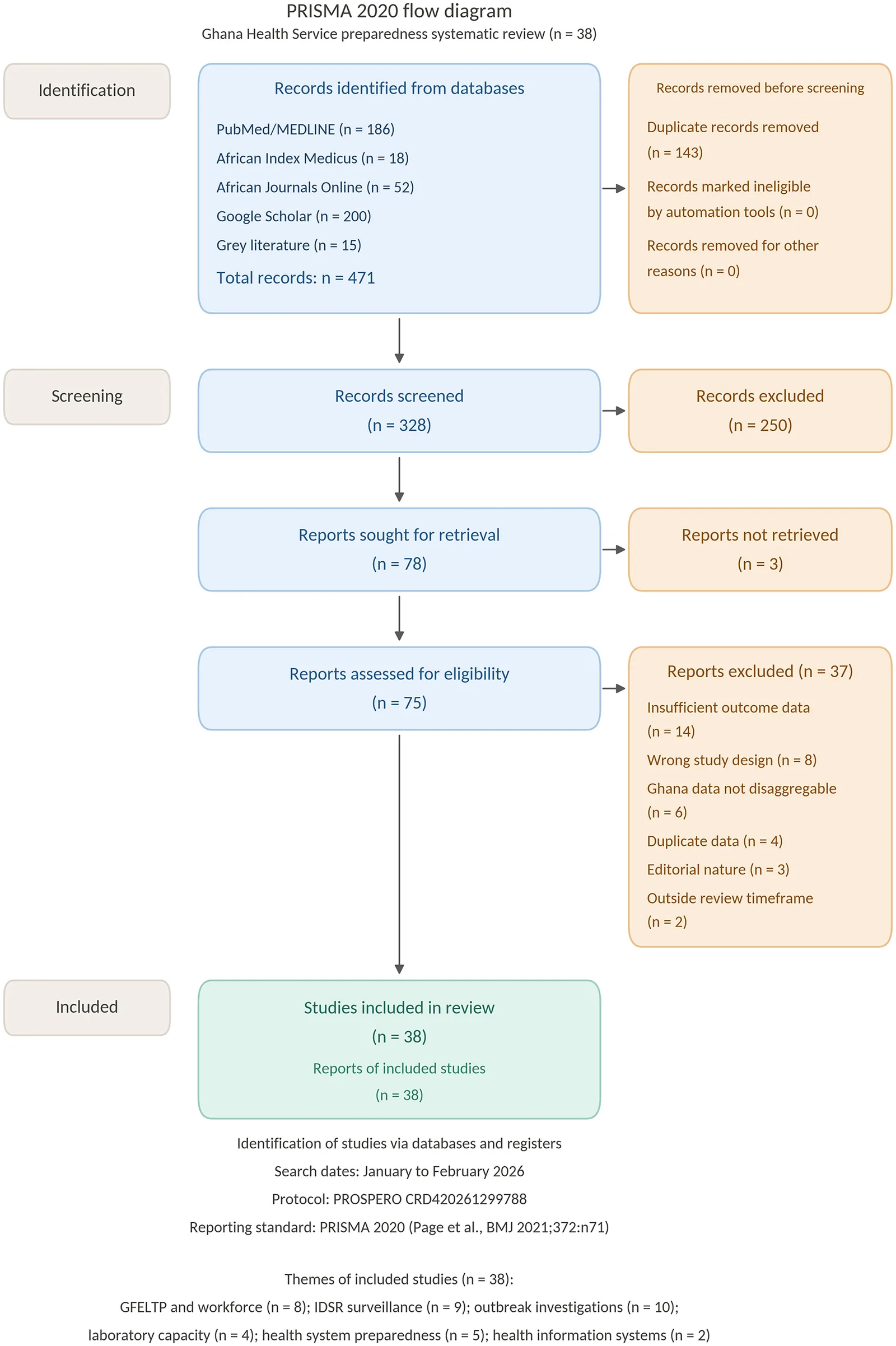
PRISMA 2020 flow diagram (24).

### 3.2 Study characteristics

Table 4 presents comprehensive characteristics of all 38 included studies. The studies span publication years 2011 to 2024, with 63.2% (*n* = 24) published from 2016 onward, reflecting increased research activity following the West African Ebola epidemic and the COVID-19 pandemic. Study designs include cross-sectional studies (*n* = 15, 39.5%), outbreak investigation reports (*n* = 10, 26.3%), surveillance system evaluations (*n* = 9, 23.7%), programme evaluations (*n* = 8, 21.1%), laboratory-based surveillance studies (*n* = 4, 10.5%), and grey literature technical reports (*n* = 2, 5.3%), with some studies contributing to multiple categories. Geographic coverage included national-level studies (*n* = 12, 31.6%), multi-regional studies (*n* = 8, 21.1%), and single-region or single-district studies (*n* = 18, 47.4%). The Greater Accra region (*n* = 11 studies) and the northern regions (*n* = 9 studies) received the greatest research attention.

**Table 4:** Systematic literature review summary of the 38 included studies.

No.	First author, year (Ref)	Design	Location	Study Period	Sample/focus	Key outcomes assessed	Key findings	Risk of bias
**Theme 1: GFELTP and workforce development (*****n*** = **8)**								
1	Wurapa et al. (45)	Programme description	National	2007–2011	First GFELTP cohorts	Programme structure, curriculum, competencies	One Health FELTP established at University of Ghana; first in West Africa; combined human and veterinary health training	Moderate
2	Ameme et al. (46)	Programme evaluative	National, regional	2007–2015	35 GFELTP graduates	Outbreak investigations (*n* = 87), surveillance contributions	87 outbreak investigations conducted; graduates contributed to surveillance system strengthening in all regions	Low
3	Ameme et al. (47)	Training programme evaluation	8 regions	2014–2015	240 frontline health workers	Training reach; knowledge gains; outbreak detection	Frontline training reached 240 workers across 8 regions; knowledge scores improved significantly	Moderate
4	Bandoh et al. (48)	Sustainability assessment	National	2007–2014	42 GFELTP graduates	Retention (68%), funding sustainability, institutionalization	68% retention rate; heavy external funding dependence; government contribution increasing	Low
5	Kenu et al. (49)	Programme review	National	2007–2017	All GFELTP graduates	Advanced (*n* = 52), intermediate (*n* = 158), frontline (*n* = 210) graduates	Total 420 graduates; represents approximately 45% of WHO-benchmarked requirement	Low
6	Lokossou et al. (50)	Regional FELTP during COVID	ECOWAS region	2020–2021	25 ECOWAS field epidemiologists	COVID-19 response role; regional epidemiologist deployment	Ghana deployed 18 of 25 (72%) ECOWAS field epidemiologists to COVID-19 response	Moderate
7	Lokossou et al. (51)	Regional workforce assessment	ECOWAS, 15 countries	2018	Regional epidemiologist workforce	Epidemiologist density; regional benchmarks; workforce gaps	Ghana had 18 field epidemiologists against benchmark of 40; 1,592 needed regionally	Moderate
8	Gebru et al. (52)	Inter-country collaboration	Sierra Leone, Ghana	2020–2022	GFELTP-Sierra Leone capacity building	Technical assistance outcomes; capacity building	GFELTP-trained residents contributed to Sierra Leone COVID-19 response; regional mentorship model documented	High
**Theme 2: IDSR surveillance systems (*****n*** = **9)**								
9	Adokiya et al. (25)	IDSR evaluation	Northern Ghana, 26 districts	2012–2013	3-region surveillance data	Completeness; timeliness; data quality	Completeness 78.3%; timeliness 52.6%; feedback from national level inconsistent	Low
10	Adokiya et al. (26)	IDSR completeness and timeliness	Northern Ghana, 43 districts	2011–2012	District and regional data	District completeness; regional completeness; timeliness	District completeness 75.8%, regional 94.2%; district timeliness 48.3%	Low
11	Tender et al. (27)	Measles surveillance evaluation	Ga West Municipality	2017	Single municipality surveillance	Simplicity; timeliness; representativeness	Simplicity rated high; timeliness 83%; representativeness limited due to facility non-reporting	Low
12	Broni et al. (28)	VHF surveillance evaluation	Bawku Municipality, Upper East	2011–2015	Border area VHF surveillance	Completeness; timeliness; PPV	Completeness 82%; timeliness 91%; PPV low due to laboratory confirmation constraint	Moderate
13	Adomako et al. (29)	TB surveillance evaluation	Ga West Municipality	2011–2016	6-year TB surveillance data	Completeness; timeliness; data quality trends	Completeness 89%; timeliness 76%; improving trends over study period	Low
14	Opoku et al. (30)	Cholera surveillance evaluation	Kumasi Metropolis, Ashanti	2020	Urban cholera surveillance	Simplicity; acceptability; timeliness	Simplicity rated high; acceptability high; timeliness 87%; surveillance response adequate during outbreak	Moderate
15	Owusu et al. (31)	TB surveillance during COVID	Single municipality	2020–2021	Pandemic impact on surveillance	Completeness decline; timeliness decline	Completeness fell from 89% to 71%; timeliness fell from 76% to 64% during COVID-19	Moderate
16	Adokiya et al. (32)	EVD surveillance preparedness	Northern Ghana, 43 districts	2014–2015	Ebola preparedness surveillance	Rapid system establishment; capacity gaps	Rapid EVD surveillance established but gaps in PPE, isolation capacity, and laboratory confirmation identified	Low
17	Mohammed et al. (33)	Malaria surveillance analysis	Sunyani Municipality, Brong-Ahafo	2020	Malaria case data analysis	Seasonal patterns; data quality	Clear seasonal malaria patterns identified; data quality adequate; analytical capacity demonstrated at district level	Moderate
**Theme 3: outbreak investigations (*****n*** = **10)**								
18	Domo et al. (35)	Meningitis outbreak investigation	Jirapa District, Upper West	2016	32 cases	Response time; attack rate; causative pathogen	Mixed pneumococcal and meningococcal outbreak confirmed; response time 3 days; attack rate 12.8/100,000	Low
19	Issahaku et al. (36)	Cholera outbreak investigation	Central Region	2016	284 cases, 4 deaths	Response delay; attack rate; CFR	Response delay 14 days; attack rate 28.4/10,000; CFR 1.4%; contaminated water source identified	Low
20	Ohene-Adjei et al. (37)	Major cholera outbreak	Greater Accra Region	2014	28,975 cases, 243 deaths	Outbreak scale; response capacity	Largest cholera outbreak in Ghana's recorded history; CFR 0.84%; response scaled over 3 months; GFELTP graduates central to investigation	Low
21	Okoh-Owusu et al. (38)	Mumps outbreak investigation	Western North Region	2022	156 cases	Late detection; surveillance gaps	Detection delayed 4 weeks; passive surveillance missed early cases; vaccine-preventable disease surveillance gaps	Moderate
22	Sarkodie et al. (39)	COVID-19 national response	National	2020–2021	National pandemic preparedness	EOC activation; testing expansion; FELTP role	Laboratory testing expanded from 2 to 43 sites within 3 months; EOC activated within 48 h of first case; GFELTP graduates led contact tracing	Low
23	Appiah et al. (40)	COVID-19 contact tracing investigation	Ablekuma North Municipality	March–April 2020	1,247 contacts traced	Secondary attack rate; lab capacity	Secondary attack rate 8.3%; laboratory turnaround time constraints affected case finding; 94.3% contact tracing completion	Moderate
24	Phebe et al. (41)	Lassa fever outbreak (cross-border)	Grand Bassa County, Liberia	2021	Cross-border GFELTP support	Regional capacity contribution	GFELTP residents contributed to inter-country investigation; cross-border collaboration documented	Moderate
25	Dzotzi et al. (42)	Bacterial enteric pathogen surveillance	2 Accra sub-metros	2014–2015	Laboratory-based surveillance	Pathogen diversity; lab capacity adequacy	Vibrio cholerae, Shigella, and Salmonella detected; sentinel laboratory capacity adequate for common enteric pathogens	Moderate
26	Ayettey et al. (43)	Aedes vector surveillance	Cape Coast	2020–2021	Entomological surveillance	Aedes density; arbovirus risk; insecticide resistance	High Aedes aegypti density confirmed; multiple insecticide resistance detected; arboviral outbreak risk quantified	Low
27	Asiedu-Bekoe et al. (44)	EVD healthcare worker preparedness	Ashanti Region	2015	384 healthcare workers surveyed	Preparedness perceptions; training gaps; PPE access	73% felt inadequately prepared; 58% reported inadequate PPE access; 65% had inadequate EVD case definition training	Low
**Theme 4: laboratory capacity (*****n*** = **4)**								
28	Opintan et al. (53)	AMR surveillance evaluation	6 sentinel labs, nationwide	2010–2013	Laboratory AMR surveillance network	Surveillance network capacity; reporting quality	AMR surveillance operational in 6 sites; culture and sensitivity testing adequate; external quality assessment needed	Low
29	Asante et al. (54)	Influenza sentinel surveillance	4 sentinel sites nationwide	2011–2019	Laboratory-based influenza surveillance	Confirmation rates; seasonal patterns	Influenza confirmation rate 18.6% among ILI cases; clear seasonal patterns with peaks March-April and July-August	Low
30	Suu-Ire et al. (55)	Viral zoonoses capacity assessment	National assessment	2019–2020	Zoonotic disease diagnostic capacity	Diagnostic capacity; pathogen coverage; surveillance gaps	No in-country testing for Lassa fever, Rift Valley fever, CCHF; limited monkeypox capacity; rabies and yellow fever testing at NMIMR	Moderate
31	Lartey et al. (56)	NMIMR research and diagnostic capacity	National reference laboratory	2017–2018	Reference laboratory capacity	Viral diagnostic scope; capacity limitations	Viral diagnostic capacity exists but limited to selected pathogens; biosafety infrastructure constrains work with high-consequence agents	High
**Theme 5: Health system preparedness (*****n*** = **5)**								
32	Asiedu-Berkoe et al. (57)	IHR core capacity assessment	National	2019	National IHR preparedness	Overall IHR capacity; domain-specific scores	Overall IHR preparedness 67%; laboratory 58%; workforce 63%; emergency preparedness 71%; AMR surveillance 83%	Low
33	Dalhat et al. (58)	ECOWAS regional preparedness	ECOWAS 15 countries	2019–2020	Regional preparedness comparison	Ghana regional ranking; strengths and gaps	Ghana ranked 3rd in ECOWAS after Nigeria and Senegal; shared regional weaknesses in emergency financing and IPC	Moderate
34	Mahama et al. (59)	Pathogen threat and surveillance analysis	National	2020–2021	Emerging pathogen risk and surveillance	Emerging pathogen surveillance gaps; One Health deficits	Current surveillance inadequate for VHFs beyond yellow fever, arboviruses, novel coronaviruses; One Health integration needed	Moderate
35	Ofori et al. (60)	NMIMR research capacity	Noguchi Memorial Institute	2017–2018	Research and surveillance capacity	Research support to GHS; collaborative surveillance	NMIMR research capacity supports GHS surveillance; collaboration with GHS documented; genomics capacity limited	High
36	WHO (61)	Joint External Evaluation	National	2017	WHO IHR core capacity evaluation	Domain-specific IHR scores	Overall score 67%; strongest: AMR (83%), zoonotic diseases (79%); weakest: radiation emergencies (42%), biosafety (50%), laboratory (58%)	Low (grey lit)
**Theme 6: Health information systems (*****n*** = **2)**								
37	GHS/DHIMS2 (34)	National HIS evaluation	National	2012–2015	DHIMS2 national implementation	Completeness trends; mobile reporting; data quality	National reporting completeness improved from 52% (2012) to 89% (2015); mobile reporting module introduced; data quality strengthens	Moderate
38	Ghana Health Service (62)	Annual surveillance reports	National	2018–2020	GHS operational surveillance data	Surveillance trends; outbreak documentation; workforce data	Surveillance data trends documented; outbreak events cataloged; GFELTP graduate deployment numbers updated	Low (grey lit)

AMR, antimicrobial resistance; CCHF, Crimean-Congo hemorrhagic fever; CFR, case fatality rate; CSM, cerebrospinal meningitis; EOC, emergency operations centre; EVD, Ebola virus disease; GHS, Ghana Health Service; IDSR, Integrated Disease Surveillance and Response; ILI, influenza-like illness; NMIMR, Noguchi Memorial Institute for Medical Research; PPE, personal protective equipment; PPV, positive predictive value; TB, tuberculosis; VHF, viral hemorrhagic fever.

### 3.3 Quality assessment results

Application of the design-specific tools described in Table 3, together with the reconciliation rules into low, moderate, and high risk-of-bias categories, produced the overall quality distribution presented in Table 5. Of 38 included studies, 19 (50.0%) were rated low risk of bias, 16 (42.1%) moderate risk of bias, and 3 (7.9%) high risk of bias.

**Table 5:** Quality assessment summary by study type.

Study type	Low risk of bias	Moderate risk of bias	High risk of bias	Total	Common quality concerns
Cross-sectional studies	5	8	2	15	Selection bias; convenience sampling; lack of comparison groups; cross-sectional design limitations
Outbreak investigations	6	4	0	10	Retrospective data quality; incomplete case ascertainment; limited follow-up
Surveillance evaluations	6	3	0	9	Single time-point assessment; data quality dependent on underlying system performance
Programme evaluations	2	4	2	8	Lack of comparison groups; self-reported outcomes; sustainability not always assessed
Laboratory studies	2	2	0	4	Limited geographic coverage; convenience sampling of sentinel sites
Grey literature	1	0	1	2	Variable peer review; organizational reporting bias
**Total**	**19**	**16**	**3**	**38**	

Bold values are the totals across the 38 included studies. The study-type rows sum to 48 because 10 studies fall into more than one design category (see Section 3.2).

### 3.4 Synthesis of findings by theme

#### 3.4.1 Surveillance system performance and capacity

Nine studies evaluated IDSR and disease-specific surveillance using standardized CDC and WHO frameworks (25–33). These studies assessed completeness, timeliness, positive predictive value, and data quality across pathogen types and health system levels.

##### 3.4.1.1 Completeness

District-level completeness in northern Ghana ranged from 75.8% to 78.3% across two studies examining 26 to 43 districts during 2011 to 2013 (25, 26). Regional-level completeness substantially exceeded district performance, reaching 94.2% in the same geographic area (26). Disease-specific surveillance evaluations found tuberculosis completeness of 89% (29), VHF surveillance completeness of 82% (28), and measles surveillance at similar levels (27). At the national level, DHIMS2 data indicate that overall reporting completeness improved from 52% in 2012 to 89% by 2015 following mobile reporting introduction and enhanced supervisory feedback (34). COVID-19 disrupted this trend, with tuberculosis surveillance completeness declining from 89% to 71% in one municipality during 2020 to 2021, alongside a timeliness decrease from 76% to 64% (31). Across the eight studies reporting quantitative completeness data, the pooled median was 82% (range 71% to 94%).

##### 3.4.1.2 Timeliness

District-level timeliness was markedly low at 48.3% to 52.6% in northern Ghana studies from 2011 to 2013 (25, 26), indicating that fewer than half of expected reports arrived within required timeframes. Disease-specific timeliness estimates were substantially higher, with VHF surveillance at 91% (28), cholera surveillance at 87% (30), measles surveillance at 83% (27), and tuberculosis surveillance at 76% (29). Commonly identified barriers to timely reporting included transportation challenges, staff shortages, competing clinical demands, inadequate supervisory feedback, and poor connectivity at peripheral facilities (25, 26). Across studies reporting quantitative timeliness data, the pooled median was 76% (range 48% to 91%).

##### 3.4.1.3 Data quality and positive predictive value

VHF surveillance demonstrated low positive predictive value despite 91% timeliness, reflecting laboratory confirmation constraints that limited surveillance specificity (28). Low positive predictive value increases the risk of false outbreak alerts and erodes trust in the surveillance system. Laboratory confirmation bottlenecks at district level constrained data quality across multiple surveillance evaluations (25, 26, 28).

These findings sit consistently within the broader sub-Saharan African pattern reported in seminal multi-country IHR analyses, in which detection capacity has improved over time but remains uneven across health system levels and disease categories (7, 8).

#### 3.4.2 Outbreak investigation capacity and response

Ten studies examined outbreak investigation methodologies and response capacity across diverse disease scenarios (35–44). These studies collectively documented substantial capacity improvements over the review period.

Response time, defined as the interval from outbreak alert to initiation of investigation, showed a dramatic reduction from 14 days during the 2016 Central Region cholera outbreak (36) to 3 days during the 2016 Jirapa meningitis outbreak (35), for comparable outbreak types investigated by GFELTP-trained field epidemiologists. This approximate five-fold improvement suggests meaningful capacity advancement following the 2014 to 2016 West African Ebola epidemic, which precipitated intensive investment in field epidemiology capacity across the ECOWAS region.

The 2014 Greater Accra cholera outbreak (28,975 cases, 243 deaths, CFR 0.84%) represents the largest cholera event in Ghana's recorded history and revealed both the destructive potential of water-borne disease in rapidly urbanizing areas and the central role of GFELTP graduates in large-scale epidemiological investigation (37). The 2020 COVID-19 response documented the Emergency Operations Center activated within 48 h of Ghana's first confirmed case on March 12, 2020, with GFELTP graduates leading contact tracing operations across affected regions (39). The Ablekuma North contact tracing investigation achieved 94.3% contact tracing completion among 1,247 contacts, with a documented secondary attack rate of 8.3%, despite laboratory turnaround time constraints (40).

Late outbreak detection persisted for lower-priority diseases. A mumps outbreak in the Western North Region in 2022 experienced a 4-week detection delay due to passive surveillance gaps for vaccine-preventable diseases not under intensified surveillance (38). This finding highlights the challenge of sustaining broad-based surveillance across all notifiable conditions.

#### 3.4.3 GFELTP programme evaluation and workforce development

Eight studies addressed GFELTP programme outcomes and workforce capacity (45–52). The GFELTP, established in 2007 as the first FELTP in West Africa, has trained 52 advanced graduates, 158 intermediate graduates, and 210 frontline graduates over the 2007 to 2017 decade, totalling approximately 420 graduates across all tiers (49). Against WHO benchmark workforce requirements for Ghana's population size, this represents approximately 45% of the field epidemiology workforce needed, with particularly critical gaps at the advanced level and in peripheral deployment (51).

The 68% graduate retention rate documented by Bandoh et al. (48) exceeds many African FELTP programmes, where rates below 50% are common. GFELTP graduates contributed to 87 documented outbreak investigations from 2007 to 2015, representing a major contribution to national outbreak response capacity (46). Frontline training reached 240 peripheral health workers across 8 regions in 2014 to 2015 alone, though total frontline programme penetration remained limited relative to the thousands of health workers requiring basic surveillance competencies (47).

Ghana's regional field epidemiology leadership is evident: Ghana possessed 18 of 25 (72%) ECOWAS field epidemiologists in 2018, and these epidemiologists were deployed across the region during COVID-19 response in 2020 to 2021 (50, 51). Ghana has required approximately 40 advanced field epidemiologists against WHO benchmarks, implying a gap of 22 epidemiologists at the advanced level alone (51). At current production rates of approximately 5 to 7 advanced graduates annually, achieving workforce sufficiency through FELTP alone would require sustained investment over two decades.

Biostatistics and epidemic modeling were not identified as core FELTP curriculum components in any of the eight programme evaluation studies (45–52), a finding with significant implications for analytical preparedness capacity.

#### 3.4.4 Laboratory capacity for infectious disease surveillance

Four studies addressed laboratory capacity across antimicrobial resistance surveillance, influenza surveillance, viral zoonoses, and reference laboratory functions (53–56).

Antimicrobial resistance surveillance operated across six sentinel sites nationally from 2010 to 2013, with culture and sensitivity testing adequate within participating facilities but external quality assessment coverage insufficient (53). Influenza sentinel surveillance across 4 sites from 2011 to 2019 demonstrated a confirmation rate of 18.6% among influenza-like illness cases, with seasonal patterns reflecting dual influenza peaks in March to April and July to August annually (54).

The viral zoonoses capacity assessment documented critical diagnostic gaps. No in-country testing capacity existed for Lassa fever, Rift Valley fever, or Crimean-Congo hemorrhagic fever; monkeypox testing capacity was established only during the 2022 outbreak; and limited PCR capacity existed for Ebola and Marburg viruses as emergency-only response rather than sustained capacity (55). Diagnostics for common pathogens including rabies (direct fluorescent antibody testing), yellow fever (serology at NMIMR), and dengue (serology at NMIMR) were available but geographically confined to Greater Accra (55, 56). COVID-19 laboratory expansion from 2 to 43 testing sites within three months demonstrated the potential for rapid scale-up when sufficient resources and political commitment are mobilized (39).

Consistent laboratory capacity constraints across all four studies included geographic concentration of advanced capacity at NMIMR in Greater Accra; insufficient sentinel site coverage for representative geographic surveillance; weak external quality assurance below reference laboratory level; inadequate biosafety infrastructure for high-consequence pathogen work; limited molecular diagnostics beyond NMIMR; and insufficient laboratory workforce across all cadres (53–56).

#### 3.4.5 Health system preparedness and IHR core capacities

Five studies assessed overall health system preparedness using IHR and other preparedness frameworks (32, 44, 57–61).

Ghana's 2017 WHO Joint External Evaluation found overall IHR core capacity at 67%, above the ECOWAS regional average (61). Strongest capacities included antimicrobial resistance surveillance (83%), zoonotic disease integration (79%), and food safety (75%). Weakest capacities included radiation emergencies (42%), biosafety and biosecurity (50%), laboratory systems (58% despite NMIMR excellence), health emergency management (63%), and human resources development (63% despite FELTP establishment) (61). A 2019 national assessment corroborated these findings, documenting overall preparedness at 67%, with workforce (63%) and laboratory (58%) remaining critical gaps (57).

Ghana ranked third among 15 ECOWAS countries for overall preparedness in a 2019 to 2020 regional comparison (58). Regional weaknesses shared with neighboring countries included inadequate emergency preparedness financing, infection prevention and control in health facilities, and risk communication systems (58). These findings are consistent with the global IHR-SPAR analyses showing that sub-Saharan African countries face persistent capacity gaps despite IHR investments, and that the 2024 IHR amendments have not yet resolved the underlying structural inequities in preparedness financing across the region (7–9).

Healthcare worker preparedness surveys revealed substantial frontline gaps despite national-level preparedness activities. Among 384 healthcare workers surveyed in Ashanti Region in 2015, 73% reported feeling inadequately prepared for EVD patient management, 58% reported inadequate PPE access, and 65% had inadequate EVD case definition training (44). This disconnect between policy-level preparedness planning and frontline operational readiness represents a recurring finding across preparedness assessments.

#### 3.4.6 Biostatistical capacity and epidemic modeling

A striking finding from this systematic review is the complete absence of studies examining biostatistical capacity or epidemic modeling capabilities in Ghana. None of the 38 included studies assessed statistical modeling for infectious diseases, epidemic forecasting, predictive analytics for outbreak detection, or workforce capacity in biostatistics and data science (25–62). This constitutes a critical evidence gap and a potential capacity gap of increasing strategic significance, as epidemic modeling underpins response planning and resource allocation for large-scale preparedness events (12, 13).

Several programme evaluations mentioned data analysis as an FELTP competency but without specification of statistical methods or modeling approaches (45, 46, 49). No study documented biostatistics training within GFELTP, statistical mentoring for residents, or modeling capacity development within the Ghana Health Service or partner institutions.

### 3.5 Assessment of risk of bias across studies

Potential sources of publication bias include geographic overrepresentation of Greater Accra and Ashanti regions (reflecting proximity to research institutions), preferential publication of successful programmes over unsuccessful initiatives, and selective publication of large or unusual outbreaks over routine events. Mitigating factors include a comprehensive search strategy incorporating grey literature and African databases, diverse funding sources without apparent commercial conflicts, and convergent findings across independent studies of different designs and settings. Funnel plots were not applicable given the narrative synthesis approach.

### 3.6 Subgroup analyses

#### 3.6.1 By health system level

Regional-level surveillance completeness (94%) substantially exceeded district-level performance (76%), consistent with resource and accountability gradients across health system levels (25, 26).

#### 3.6.2 By time period

Temporal trends indicated performance improvements over the review period. National DHIMS2 completeness increased from 52% (2012) to 89% (2015) (34); outbreak response times decreased approximately five-fold across comparable outbreaks investigated in 2016 (35, 36); and GFELTP graduate numbers grew steadily from 10 (2007–2010) to 52 advanced graduates by 2017 (49). COVID-19 disrupted upward trends, with surveillance completeness declining and laboratory systems briefly overwhelmed.

#### 3.6.3 By disease type

High-consequence pathogens receiving dedicated resources after the 2014 Ebola epidemic (VHFs, COVID-19) demonstrated better surveillance metrics and faster response times than endemic diseases with lower political profile (mumps, routine tuberculosis) (31, 38).

## 4 Discussion

### 4.1 Summary of main findings

This systematic review synthesized 38 studies examining Ghana Health Service preparedness for field epidemiology and applied biostatistics across surveillance systems, outbreak investigation, workforce development, laboratory capacity, and health system preparedness in studies published between 2011 and 2024. Findings reveal advancing but incomplete preparedness against the WHO IHR core capacity framework.

Surveillance systems demonstrated moderate performance, with median completeness of 82% and timeliness of 76% indicating that approximately one-fifth of expected reports were missing or delayed. The pronounced gradient between regional-level completeness (94%) and district-level performance (76%) underscores that strengthening efforts must prioritize peripheral levels where early detection is most critical. COVID-19's disruption of routine surveillance, reducing completeness by 18 percentage points in one assessed municipality, demonstrated system fragility under competing demands (31). Outbreak response capacity showed encouraging improvement, with response times decreasing from 14 days for the 2016 Central Region cholera outbreak to 3 days for the 2016 Jirapa meningitis outbreak for comparable outbreak types, and GFELTP graduates contributing to 87 documented outbreak investigations (46). Capacity remained insufficient in some areas, particularly for vaccine-preventable diseases where surveillance weakened, with a four-week mumps outbreak detection delay documented in 2022 (38).

The GFELTP programme is Ghana's strongest preparedness asset, producing 420 graduates across three training tiers from 2007 to 2017 (49). The 68% retention rate exceeds many African FETPs (48). Ghana's possession of 72% of ECOWAS field epidemiologists demonstrates regional leadership (50, 51). Critical gaps remain in workforce density (45% of WHO benchmark), geographic distribution, frontline programme penetration, and the complete absence of biostatistics training within the curriculum. Laboratory capacity is a rate-limiting constraint, concentrated in 2 to 6 sentinel sites depending on pathogen type, with absent or extremely limited capacity for most emerging and zoonotic pathogens (55). COVID-19 demonstrated that capacity can be scaled rapidly with sufficient resources, but sustainability of expanded capacity remains uncertain.

Health system preparedness achieved moderate levels (67% overall IHR core capacity) with deficits in laboratory systems (58%), workforce (63%), and emergency financing (57, 61). Healthcare worker preparedness surveys documented 73% of frontline workers feeling inadequately prepared for high-consequence pathogen response (44), indicating a disconnect between national-level policy planning and facility-level operational readiness. Biostatistical and epidemic modeling capacity represents the most striking gap: no study among the 38 included addressed statistical modeling, predictive analytics, or biostatistical capacity development, and no evidence emerged of dedicated modeling capacity within the Ghana Health Service or partner institutions.

### 4.2 Interpretation of findings

The Ghana evidence base reviewed here sits within and largely confirms the global pattern documented by seminal IHR-based analyses. Kandel and colleagues found that approximately half of countries lacked operational readiness for high-impact respiratory pathogens, with sub-Saharan Africa most affected (7). Eze and colleagues showed that even between 2018 and 2022 SPAR cycles, capacity gaps persisted across all five IHR domains for sub-Saharan African countries (8). The recent IHR 2024 amendments analyzed by Gostin and colleagues, while introducing important regulatory changes, do not by themselves address the underlying capacity and financing inequities that this review documents in concrete operational terms within Ghana (9).

GFELTP's substantial impact validates the FETP model's effectiveness in Africa, consistent with evidence from other countries where FETPs build investigation and response capacity when graduates are appropriately deployed (11). Yet even successful programmes produce graduates too slowly to rapidly close workforce gaps. At current production rates, Ghana requires sustained investment over two decades to achieve WHO-recommended workforce density without accounting for attrition. Ghana's three-tiered approach combining advanced, intermediate, and frontline training represents a pragmatic compromise, though frontline programme penetration remains limited relative to need. Graduate geographic concentration in Greater Accra reflects a common challenge in human resources for health globally. Without deployment incentives or requirements, graduates gravitate toward urban centers offering better professional opportunities (48, 51). Career pathway challenges compound this, as graduates progress to administrative positions with less direct epidemiological practice. The retention rate of 68%, while above regional average (48), means that almost one-third of GFELTP investment exits active epidemiological practice, warranting structured career pathways maintaining epidemiological practice across career stages.

Ghana's moderate surveillance performance despite two decades of investment reflects a common pattern in resource-limited settings, where surveillance system strengthening requires sustained operational support beyond initial training and infrastructure investment (25, 26). The performance gradient across health system levels suggests differentiated strengthening strategies are needed rather than uniform interventions. Regional positions attract stronger staff, benefit from greater supervision intensity, and have better connectivity. District and facility levels require targeted operational strengthening addressing transportation, feedback, and motivation. COVID-19 disruption of routine surveillance illustrates inevitable trade-offs when health systems face overwhelming competing demands simultaneously (31, 39).

Laboratory capacity emerged as a rate-limiting factor for multiple epidemic preparedness functions. The concentration of advanced capacity at NMIMR reflects the typical pattern in resource-limited settings where economies of scale and expertise concentration favor centralization, yet centralization creates geographic access challenges (53–56). COVID-19's rapid laboratory expansion from 2 to 43 sites demonstrated feasibility of rapid scale-up with resources and political will, though sustainability post-pandemic requires transitioning COVID infrastructure to multi-pathogen capacity rather than allowing contraction (39).

The complete absence of biostatistics and epidemic modeling evidence represents a strategic gap with increasingly significant implications. Modern epidemic preparedness depends on real-time data analysis, statistical anomaly detection, epidemic forecasting, mathematical modeling of intervention effectiveness, and genomic epidemiology for transmission tracking (12, 13). COVID-19 highlighted modeling's value globally; however, most African countries, including Ghana, lacked indigenous modeling capacity and depended on external modelers, creating challenges with African context calibration, delays in outputs when needed for time-sensitive decisions, and limited local ownership of model assumptions (13). Building indigenous capacity addresses these limitations while developing a cadre of technical experts bridging epidemiology and quantitative sciences.

### 4.3 Limitations

This review has several limitations that must be acknowledged alongside its strengths. First, publication bias is suspected, with potential overrepresentation of Greater Accra and Ashanti regions, successful programmes, and large outbreak investigations in the published literature. Second, the language restriction to English potentially excludes relevant publications from Francophone neighboring countries that could have provided broader regional context. Third, database access limitations prevented searching Embase, Web of Science, and Scopus due to subscription cost constraints, though African-specific databases partially compensated for this. Fourth, substantial heterogeneity across study designs, populations, and outcome definitions precluded quantitative meta-analysis and limited the precision of summary estimates. Fifth, three studies rated high risk of bias may have influenced findings, though convergence across independent studies suggests robust conclusions. Sixth, proxy bias is an additional limitation, in that many included studies used surveillance data completeness and timeliness as proxies for preparedness capacity, which may not fully capture actual epidemiological response capability. Seventh, heterogeneity bias is present in that the wide variation in study designs, geographic focus, and time periods limits direct comparability across studies even where similar metrics are reported. These limitations are inherent to narrative synthesis methodology and the diversity of evidence available for resource-limited settings.

### 4.4 Implications for policy and practice

Based on the synthesized evidence, six priority areas merit action.

First, GFELTP expansion should be aggressively pursued as Ghana's highest-impact preparedness investment. Recommendations include increasing advanced programme intake to 10 to 15 trainees per cohort, dramatically expanding frontline training to reach thousands of peripheral health workers, implementing deployment policies ensuring service in high-need districts, integrating biostatistics and epidemic modeling as core curriculum competencies, and diversifying funding to reduce external dependence (48, 49, 51).

Second, surveillance system strengthening requires differentiated strategies by health system level. District and facility-level priorities include intensifying supervisory visits, enhancing feedback through dashboards and responsive actions demonstrating data use, addressing transportation barriers, and providing performance incentives for consistent high-quality reporting (25, 26). National priorities include real-time data visualization, regular data quality audits, strengthened event-based surveillance, and DHIMS2 platform sustainability investment (34).

Third, laboratory network expansion should prioritize geographic decentralization through regional testing hubs, point-of-care testing strategies for selected pathogens, investment in sequencing and genomics capacity, comprehensive external quality assessment, and sustainable operational financing (53–56).

Fourth, biostatistics and epidemic modeling capacity development should be pursued through establishing a modeling unit within the Ghana Health Service or partner institution, integrating advanced statistical and modeling training into GFELTP, training dedicated biostatisticians through targeted scholarships with return-of-service agreements, and developing partnerships with academic modeling centers globally (12, 13).

Fifth, sustainable preparedness financing through dedicated government budget allocations, epidemic preparedness funds with rapid access mechanisms, and multi-year financing commitments is essential to replace reactive outbreak response funding with proactive preparedness investment (58, 61).

Sixth, frontline healthcare worker preparedness should shift from training workshop models toward hands-on practical training, facility-level PPE stockpiling, regular simulations and drills, and clear point-of-care protocols, addressing the documented disconnect between national planning and frontline operational readiness (32, 44).

### 4.5 Conclusions

This systematic review, synthesizing 38 studies published between 2011 and 2024, documents advancing but incomplete Ghana Health Service preparedness for field epidemiology and applied biostatistics. GFELTP has established Ghana as a regional field epidemiology leader, producing 420 graduates contributing to surveillance, outbreak investigation, and pandemic response. Outbreak response times have improved dramatically. Laboratory capacity has expanded. Yet workforce density remains at 45% of benchmark requirements, surveillance timeliness at peripheral levels falls below 55%, laboratory networks remain geographically concentrated, biostatistical modeling capacity is essentially absent, and emergency financing mechanisms remain inadequate.

Closing these gaps requires sustained political commitment, adequate resource allocation, workforce expansion at all tiers, differentiated surveillance strengthening by health system level, laboratory network decentralization, and development of indigenous biostatistics and epidemic modeling capacity as an emerging strategic priority. The ongoing Ebola outbreak in the Democratic Republic of Congo at the time of writing serves as a continuing reminder that regional preparedness gains can be rapidly tested. Ghana's experience provides broadly applicable lessons for resource-limited settings globally pursuing field epidemiology capacity development within the WHO IHR core capacity framework.

## Data Availability

The original contributions presented in the study are included in the article/Supplementary material, further inquiries can be directed to the corresponding author.
