# Supplementary material for: Preparedness of the Ghana Health Service for field epidemiology and applied biostatistics: a systematic review of infectious disease surveillance, outbreak investigation methodologies, and statistical modeling capacities in resource-limited settings
Source: Front Public Health. 2026 Sep 16;14:1814212. doi: 10.3389/fpubh.2026.1814212 (PMC13624072; doi:10.3389/fpubh.2026.1814212)
Supplement: Supplementary file 1 [file Table_1.docx]

# SUPPLEMENTARY FILE 1: PRISMA 2020 CHECKLIST

**Manuscript Title:** Preparedness of the Ghana Health Service for Field Epidemiology and Applied Biostatistics: A Systematic Review of Infectious Disease Surveillance, Outbreak Investigation Methodologies, and Statistical Modeling Capacities in Resource-Limited Settings

**Corresponding Author:** Victor Luckyboy Dzramado

**Journal:** Frontiers in Public Health — Infectious Diseases: Epidemiology and Prevention

**PROSPERO Registration:** CRD420261299788

**Reporting Guideline:** Page MJ, McKenzie JE, Bossuyt PM, Boutron I, Hoffmann TC, Mulrow CD, et al. The PRISMA 2020 statement: an updated guideline for reporting systematic reviews. BMJ. 2021;372:n71. doi: 10.1136/bmj.n71

**PRISMA 2020 CHECKLIST OF ITEMS TO INCLUDE WHEN REPORTING A SYSTEMATIC REVIEW**

| **Section / Topic** | **Item #** | **Checklist Item** | **Location in Manuscript** | **Reported?** |
| --- | --- | --- | --- | --- |
| **TITLE** |  |  |  |  |
| Title | 1 | Identify the report as a systematic review. | Title page; the title explicitly identifies the work as "A Systematic Review of Infectious Disease Surveillance, Outbreak Investigation Methodologies, and Statistical Modeling Capacities in Resource-Limited Settings." | Yes |
| **ABSTRACT** |  |  |  |  |
| Abstract | 2 | See the PRISMA 2020 for Abstracts checklist (Page MJ et al., BMJ 2021;372:n71, Table 2). The Abstract includes Background and Research Question, Methods, Results, and Conclusion, with structured reporting of the 38 included studies, search dates, screening figures, key quantitative findings (surveillance completeness median 82%, timeliness median 76%, GFELTP graduate numbers, outbreak response times, IHR core capacity scores), and conclusion. | Abstract (page 1 of manuscript). | Yes |
| **INTRODUCTION** |  |  |  |  |
| Rationale | 3 | Describe the rationale for the review in the context of existing knowledge. | Section 1.1 (Global Context and Seminal Work on IHR Core Capacities) and Section 1.2 (The Ghana Context), citing seminal IHR-based multi-country analyses (Kandel et al., 2020; Eze et al., 2024; Gostin et al., 2025) and identifying the absence of a comprehensive Ghana-specific synthesis as the rationale. | Yes |
| Objectives | 4 | Provide an explicit statement of the objective(s) or question(s) the review addresses. | Section 1.4 (Primary Research Question and Objectives) presents the primary research question and six secondary objectives explicitly. | Yes |
| **METHODS** |  |  |  |  |
| Eligibility criteria | 5 | Specify the inclusion and exclusion criteria for the review and how studies were grouped for the syntheses. | Section 2.2 (Eligibility Criteria — PICOS Framework with Ghana-Specific Disaggregation Requirement) and Table 1 specify all inclusion and exclusion criteria across Population, Interventions/Exposures, Comparators, Outcomes (primary and secondary), Study Designs, Setting, Time Period, and Language. Studies were grouped for synthesis across six pre-specified thematic areas defined in Section 2.7. | Yes |
| Information sources | 6 | Specify all databases, registers, websites, organisations, reference lists, and other sources searched or consulted to identify studies. Specify the date when each source was last searched or consulted. | Section 2.3 (Information Sources and Search Strategy) and Table 2. Four electronic databases were searched (PubMed/MEDLINE 15 Jan 2026; African Index Medicus 16 Jan 2026; AJOL 17 Jan 2026; Google Scholar 18-20 Jan 2026). Grey literature sources are explicitly named in Table 2 (WHO JEE reports for Ghana, Ghana Health Service annual reports, GFELTP programme evaluations, IDSR assessment reports, outbreak investigation reports, WAHO regional assessments, AFENET publications, and institutional websites of GHS, University of Ghana School of Public Health, NMIMR, and CDC Africa). All searches were completed by 30 Jan 2026. | Yes |
| Search strategy | 7 | Present the full search strategies for all databases, registers, and websites, including any filters and limits used. | Section 2.3 and Table 2 present search strategies for each database, including the Boolean terms used, MeSH application in PubMed, search date, and number of records retrieved. Full search strategies for each database are also available as separate supplementary documents (PubMed Search Strategy, African Index Medicus Search Strategy, AJOL Search Strategy, Google Scholar Comprehensive Search, and Google Scholar Grey Literature Specific Searches). Filters applied: 2000-2026, English language. | Yes |
| Selection process | 8 | Specify the methods used to decide whether a study met the inclusion criteria of the review, including how many reviewers screened each record and each report retrieved, whether they worked independently, and if applicable, details of automation tools used in the process. | Section 2.4 (Study Selection Process). Two reviewers (VLD and OUL) independently conducted title and abstract screening and full-text assessment using Covidence systematic review software. Inter-rater agreement: kappa = 0.82 at title/abstract stage and kappa = 0.76 at full-text stage. Discrepancies resolved through consensus discussion. | Yes |
| Data collection process | 9 | Specify the methods used to collect data from reports, including how many reviewers collected data from each report, whether they worked independently, any processes for obtaining or confirming data from study investigators, and if applicable, details of automation tools used in the process. | Section 2.5 (Data Collection and Extraction). Both reviewers independently extracted data from all 38 included studies using a comprehensive data extraction form that was pilot tested on five studies and refined. Twelve discrepancies were resolved by re-examination of source articles and consensus discussion. No automation tools were used for data extraction. | Yes |
| Data items | 10a | List and define all outcomes for which data were sought. Specify whether all results that were compatible with each outcome domain in each study were sought (e.g., for all measures, time points, analyses), and if not, the methods used to decide which results to collect. | Section 2.2 and Table 1 define all outcomes (two primary: surveillance system performance indicators, outbreak response metrics; three secondary: workforce capacity indicators, laboratory capacity indicators, preparedness assessment scores). All results compatible with each outcome domain were sought from each included study. | Yes |
| Data items | 10b | List and define all other variables for which data were sought (e.g., participant and intervention characteristics, funding sources). Describe any assumptions made about any missing or unclear information. | Section 2.5. Variables extracted included study characteristics (author, year, design, location, study period), population characteristics, methodological details, intervention and surveillance system descriptions, funding sources where reported, and outcome metrics. Where information was missing or unclear, this was noted in the extraction form; no imputation or assumption-based estimation was performed. | Yes |
| Study risk of bias assessment | 11 | Specify the methods used to assess risk of bias in the included studies, including details of the tool(s) used, how many reviewers assessed each study and whether they worked independently, and if applicable, details of automation tools used in the process. | Section 2.6 (Quality Assessment) and Table 3 specify the design-specific tools applied: Newcastle-Ottawa Scale adapted for cross-sectional studies (Wells et al.); CDC Updated Guidelines (German et al., MMWR 2001); RE-AIM framework (Glasgow et al., AJPH 1999); Outbreak Investigation Methodology Checklist adapted from WHO/CDC standards; AACODS checklist (Tyndall, Flinders University, 2010). Two reviewers independently assessed all 38 studies. Inter-rater agreement: kappa = 0.76. Six disagreements resolved by consensus. No automation tools used. | Yes |
| Effect measures | 12 | Specify for each outcome the effect measure(s) (e.g., risk ratio, mean difference) used in the synthesis or presentation of results. | Section 2.7 and throughout Section 3.4. As narrative synthesis was used given heterogeneity, effect measures were not pooled. Presented metrics include proportions and percentages (surveillance completeness, timeliness, PPV, IHR core capacity scores), absolute counts (graduate numbers, outbreak cases, deaths), durations (response time in days), and case fatality rates. Medians and ranges were calculated where comparable metrics across studies were available. | Yes |
| Synthesis methods | 13a | Describe the processes used to decide which studies were eligible for each synthesis (e.g., tabulating the study intervention characteristics and comparing against the planned groups for each synthesis). | Section 2.7. Extracted data were organised across six pre-specified thematic areas: (1) surveillance system performance; (2) outbreak investigation capacity; (3) GFELTP and workforce development; (4) laboratory capacity; (5) biostatistical capacity and epidemic modeling; (6) health system preparedness and IHR core capacities. Studies were tabulated within each theme (Table 4) and synthesised narratively within each theme in Section 3.4. | Yes |
| Synthesis methods | 13b | Describe any methods required to prepare the data for presentation or synthesis, such as handling of missing summary statistics, or data conversions. | Section 2.7. No statistical conversions were performed given the narrative synthesis approach. Missing data were reported as missing rather than imputed. Quantitative metrics were tabulated as reported in source studies. | Yes |
| Synthesis methods | 13c | Describe any methods used to tabulate or visually display results of individual studies and syntheses. | Section 2.7. Tabulation was the primary method for visual display, as presented in Table 4 (38-study summary), Table 5 (quality assessment summary), and within-text quantitative summaries by theme. Subgroup analyses by health system level, time period, and disease type were presented in Section 3.6. | Yes |
| Synthesis methods | 13d | Describe any methods used to synthesise results and provide a rationale for the choice(s). If meta-analysis was performed, describe the model(s), method(s) to identify the presence and extent of statistical heterogeneity, and software package(s) used. | Section 2.7. Narrative synthesis was selected as the primary analytic approach because substantial heterogeneity across study designs, populations, outcome definitions, and time periods precluded meta-analysis. Meta-analysis would have been considered only if three or more studies had reported comparable outcomes using compatible methods and populations; this criterion was not met for any outcome domain. No meta-analytic model, statistical heterogeneity assessment, or statistical software was therefore applied. | Yes |
| Synthesis methods | 13e | Describe any methods used to explore possible causes of heterogeneity among study results (e.g., subgroup analysis, meta-regression). | Section 3.6 (Subgroup Analyses) presents narrative subgroup analyses by health system level (national, regional, district, facility), time period (pre-2014, 2014-2019, 2020-2024), and disease type (high-consequence pathogens vs endemic diseases vs vaccine-preventable diseases). Meta-regression was not performed. | Yes |
| Synthesis methods | 13f | Describe any sensitivity analyses conducted to assess robustness of the synthesised results. | Sensitivity analyses were not formally pre-specified or conducted given the narrative synthesis design. The robustness of conclusions was supported by convergence of findings across multiple independent studies with different designs and settings, as discussed in Section 3.5 (Assessment of Risk of Bias Across Studies). | Yes (limitation acknowledged) |
| Reporting bias assessment | 14 | Describe any methods used to assess risk of bias due to missing results in a synthesis (arising from reporting biases). | Section 3.5 (Assessment of Risk of Bias Across Studies). Potential publication bias was assessed narratively, with discussion of geographic overrepresentation of Greater Accra and Ashanti regions, preferential publication of successful programmes, and selective publication of large outbreaks. Funnel plots were not applicable given the narrative synthesis approach. Mitigation included comprehensive search of African databases and grey literature. | Yes |
| Certainty assessment | 15 | Describe any methods used to assess certainty (or confidence) in the body of evidence for an outcome. | Section 2.6 and Section 3.3. Certainty was assessed through the design-specific quality assessment tools (Table 3) with explicit reconciliation rules into low, moderate, and high risk-of-bias categories. The overall distribution (12 low risk, 21 moderate risk, 5 high risk) is presented in Table 5. Note that GRADE was considered but not applied to this review given the predominantly narrative, mapping-style nature of the evidence base, as discussed in the manuscript. | Yes |
| **RESULTS** |  |  |  |  |
| Study selection | 16a | Describe the results of the search and selection process, from the number of records identified in the search to the number of studies included in the review, ideally using a flow diagram. | Section 3.1 (Study Selection) and Figure 1 (PRISMA 2020 Flow Diagram). 471 records identified (PubMed 186, AIM 18, AJOL 52, Google Scholar 200, grey literature 15); 143 duplicates removed; 328 unique records screened; 250 excluded at title/abstract; 78 records assessed for full-text retrieval; 75 retrieved; 37 excluded at full-text; 38 studies included. | Yes |
| Study selection | 16b | Cite studies that might appear to meet the inclusion criteria, but which were excluded, and explain why they were excluded. | Section 2.4 documents reasons for exclusion at full-text stage with counts: insufficient outcome data (n=14), wrong study design (n=8), Ghana-specific data not disaggregable (n=6), duplicate data (n=4), editorial nature (n=3), data collection period outside the review's timeframe (n=2). A full list of excluded studies with reasons is available from the corresponding author upon reasonable request. | Yes |
| Study characteristics | 17 | Cite each included study and present its characteristics. | Section 3.2 and Table 4 (Systematic Literature Review Summary of the 38 Included Studies). Table 4 presents, for each of the 38 included studies: first author and year, design, location, study period, sample/focus, key outcomes assessed, key findings, and risk of bias rating. Studies are organised across six thematic groupings. | Yes |
| Risk of bias in studies | 18 | Present assessments of risk of bias for each included study. | Risk of bias ratings (Low, Moderate, or High) are presented for each of the 38 studies in the final column of Table 4 (Section 3.2). The summary distribution is presented in Table 5 (Section 3.3). | Yes |
| Results of individual studies | 19 | For all outcomes, present, for each study: (a) summary statistics for each group (where appropriate), and (b) an effect estimate and its precision (e.g., confidence interval), ideally using structured tables or plots. | Table 4 presents, for each of the 38 included studies, the key outcomes assessed and the key findings as reported in the source study. Quantitative metrics (surveillance completeness percentages, timeliness percentages, graduate numbers, outbreak case counts, response times, CFRs, IHR scores) are presented as reported in source studies. Confidence intervals are reported where available in source studies but were not consistently provided across all studies; this is acknowledged as a limitation. | Yes |
| Results of syntheses | 20a | For each synthesis, briefly summarise the characteristics and risk of bias among contributing studies. | Section 3.4 (Synthesis of Findings by Theme), with each of the six subsections (3.4.1 through 3.4.6) opening with the number of contributing studies and a brief characterisation. Risk of bias for contributing studies is summarised across each theme by reference to Table 4. | Yes |
| Results of syntheses | 20b | Present results of all statistical syntheses conducted. If meta-analysis was done, present for each the summary estimate and its precision (e.g., confidence interval) and measures of statistical heterogeneity. If comparing groups, describe the direction of the effect. | Section 3.4. Quantitative results within each synthesis are presented as medians, ranges, percentages, and absolute counts where comparable across studies (e.g., surveillance completeness pooled median 82% with range 71% to 94%; timeliness pooled median 76% with range 48% to 91%; outbreak response time improvement from 14 days to 3 days). No meta-analytic summary estimates were calculated, as explained in Item 13d. | Yes |
| Results of syntheses | 20c | Present results of all investigations of possible causes of heterogeneity among study results. | Section 3.6 (Subgroup Analyses) presents narrative subgroup findings by health system level, time period, and disease type. Heterogeneity by health system level was the most pronounced finding (regional completeness 94% vs district completeness 76%). | Yes |
| Results of syntheses | 20d | Present results of all sensitivity analyses conducted to assess the robustness of the synthesised results. | Formal sensitivity analyses were not conducted, as noted in Item 13f. Robustness is discussed in Section 3.5 and Section 4.3 (Limitations) through reference to convergence of findings across multiple independent studies. | Yes (limitation acknowledged) |
| Reporting biases | 21 | Present assessments of risk of bias due to missing results (arising from reporting biases) for each synthesis assessed. | Section 3.5 (Assessment of Risk of Bias Across Studies) presents narrative assessment of potential publication bias for each synthesis area. Funnel plots were not applicable given narrative synthesis. | Yes |
| Certainty of evidence | 22 | Present assessments of certainty (or confidence) in the body of evidence for each outcome assessed. | Section 3.3 and Table 5 present quality distributions across studies. Per Reviewer 5's guidance, GRADE certainty ratings were not applied to this review given the predominantly narrative and mapping nature of the evidence base; this is explicitly discussed in the Methods and Limitations. | Yes (modified approach explained) |
| **DISCUSSION** |  |  |  |  |
| Discussion | 23a | Provide a general interpretation of the results in the context of other evidence. | Section 4.1 (Summary of Main Findings) and Section 4.2 (Interpretation of Findings). The interpretation explicitly situates Ghana findings within the broader sub-Saharan African pattern documented by the seminal IHR-based multi-country analyses (Kandel et al., 2020; Eze et al., 2024; Gostin et al., 2025). | Yes |
| Discussion | 23b | Discuss any limitations of the evidence included in the review. | Section 4.3 (Limitations) discusses seven specific limitations, including publication bias, English-language restriction, database access constraints, heterogeneity precluding meta-analysis, presence of five high-risk-of-bias studies, proxy bias, and heterogeneity bias. | Yes |
| Discussion | 23c | Discuss any limitations of the review processes used. | Section 4.3 (Limitations) addresses review process limitations including database coverage (Embase, Web of Science, and Scopus not searched due to subscription constraints), English-only inclusion, and narrative synthesis approach rather than meta-analysis. | Yes |
| Discussion | 23d | Discuss implications of the results for practice, policy, and future research. | Section 4.4 (Implications for Policy and Practice) presents six numbered priority areas with detailed evidence-based recommendations: GFELTP expansion, surveillance strengthening, laboratory network expansion, biostatistics and epidemic modeling capacity development, sustainable preparedness financing, and frontline healthcare worker preparedness. Section 4.5 (Conclusions) addresses future research implications. | Yes |
| **OTHER INFORMATION** |  |  |  |  |
| Registration and protocol | 24a | Provide registration information for the review, including the register name and registration number, or state that the review was not registered. | Title page and Section 2.1. The review was prospectively registered with PROSPERO on 3 February 2026 (Registration Number CRD420261299788). | Yes |
| Registration and protocol | 24b | Indicate where the review protocol can be accessed, or state that a protocol was not prepared. | Title page and Section 2.1. The protocol is publicly accessible at <https://www.crd.york.ac.uk/PROSPERO/view/CRD420261299788>. | Yes |
| Registration and protocol | 24c | Describe and explain any amendments to information provided at registration or in the protocol. | Section 2.1 states that no substantial protocol deviations occurred during the conduct of the review. Minor refinements made during peer review (reduction of outcomes from 13 to 5 in response to reviewer feedback, and explicit operationalisation of biostatistical modeling capacity indicators) are documented in the Methods and in the response to reviewers. | Yes |
| Support | 25 | Describe sources of financial or non-financial support for the review, and the role of the funders or sponsors in the review. | Declarations section (Funding subsection). This systematic review received no specific funding from any public, commercial, or not-for-profit funding agency. Institutional support was provided by the University of Cape Coast Department of Health Information Management and Cape Coast Teaching Hospital. No external funders influenced study design, data collection, analysis, interpretation, or manuscript preparation. | Yes |
| Competing interests | 26 | Declare any competing interests of review authors. | Declarations section (Conflicts of Interest subsection). The authors declare no conflicts of interest. No author has financial or professional relationships that could inappropriately influence this systematic review. | Yes |
| Availability of data, code, and other materials | 27 | Report which of the following are publicly available and where they can be found: template data collection forms; data extracted from included studies; data used for all analyses; analytic code; any other materials used in the review. | Declarations section (Data Availability Statement). The data supporting this review's findings are available within the article and supplementary materials. The complete extracted dataset from all 38 included studies is available from the corresponding author upon reasonable request. The protocol is publicly accessible via PROSPERO (CRD420261299788). The PRISMA 2020 checklist is provided as this Supplementary File 1. No analytic code was developed as no quantitative meta-analysis was conducted. | Yes |

**DECLARATION OF CONFORMANCE**

The authors confirm that this systematic review has been reported in accordance with the PRISMA 2020 statement (Page MJ, McKenzie JE, Bossuyt PM, Boutron I, Hoffmann TC, Mulrow CD, et al. The PRISMA 2020 statement: an updated guideline for reporting systematic reviews. BMJ 2021;372:n71). All 27 items of the PRISMA 2020 checklist have been addressed in the manuscript, with locations of each item documented in the table above. The PRISMA 2020 Flow Diagram (Figure 1 in the manuscript) presents the search and selection process in accordance with the standard template available at <http://prisma-statement.org/prismastatement/flowdiagram.aspx>.

**Corresponding author confirmation:**

Victor Luckyboy Dzramado
Department of Biostatistics, Cape Coast Teaching Hospital
Cape Coast, Ghana
Email: [mldzramado@st.knust.edu.gh](mailto:mldzramado@st.knust.edu.gh)

**On behalf of all co-authors:** Obed U. Lasim, William Wilberforce Amoah, Samuel Antwi, Joana Edem Koto, and Doris Hagan.

**Date:** June 2026

**Reference for the PRISMA 2020 Statement:**

Page MJ, McKenzie JE, Bossuyt PM, Boutron I, Hoffmann TC, Mulrow CD, Shamseer L, Tetzlaff JM, Akl EA, Brennan SE, Chou R, Glanville J, Grimshaw JM, Hróbjartsson A, Lalu MM, Li T, Loder EW, Mayo-Wilson E, McDonald S, McGuinness LA, Stewart LA, Thomas J, Tricco AC, Welch VA, Whiting P, Moher D. The PRISMA 2020 statement: an updated guideline for reporting systematic reviews. BMJ. 2021;372:n71. doi: 10.1136/bmj.n71

**Reference for the PRISMA 2020 for Abstracts Checklist (Item 2):**

Page MJ, McKenzie JE, Bossuyt PM, et al. The PRISMA 2020 statement: an updated guideline for reporting systematic reviews. BMJ 2021;372:n71. Table 2: PRISMA 2020 for Abstracts checklist.
